# Transcriptome-Based Identification and Molecular Evolution of the *Cytochrome P450* Genes and Expression Profiling under Dimethoate Treatment in Amur Stickleback (*Pungitius sinensis*)

**DOI:** 10.3390/ani9110873

**Published:** 2019-10-28

**Authors:** Jun Cao, Xiuzhu Cheng

**Affiliations:** Institute of Life Sciences, Jiangsu University, Zhenjiang 212013, China

**Keywords:** CYP, Gasterosteus, selective pressure, gene duplication, immunotoxicity, detoxication

## Abstract

**Simple Summary:**

Cytochrome P450s (CYPs) are a family of membrane-bound monooxygenase proteins. In this study, 58 *CYP* genes were identified in Amur stickleback (*Pungitius sinensis*). Motif distribution, recombination, and selection were performed to investigate their evolutionary history. In addition, expression profiles of *CYPs* were examined following dimethoate treatment. The results will provide a useful reference for further functional analyses.

**Abstract:**

Cytochrome P450s (CYPs) are a family of membrane-bound mono-oxygenase proteins, which are involved in cell metabolism and detoxification of various xenobiotic substances. In this study, we identified 58 putative *CYP* genes in Amur stickleback (*Pungitius sinensis*) based on the transcriptome sequencing. Conserved motif distribution suggested their functional relevance within each group. Some present recombination events have accelerated the evolution of this gene family. Moreover, a few positive selection sites were identified, which may have accelerated the functional divergence of this family of proteins. Expression patterns of these *CYP* genes were investigated and indicated that most were affected by dimethoate treatment, suggesting that *CYPs* were involved in the detoxication of dimethoate. This study will provide a foundation for the further functional investigation of *CYP* genes in fishes.

## 1. Introduction

Cytochrome P450s (CYPs) are a family of membrane-bound hemoproteins that catalyze a variety of mono-oxygenase reactions, which play key roles in detoxification and cellular metabolism of xenobiotics [1]. The name comes from its discovery as a pigment that absorbs 450 nm wavelength of light in a complex with carbon monoxide. The maximum absorption of CYP is 420 nm in inactive form [2,3]. CYPs are divided into different families and subfamilies based on their special function and species existence. Different families are represented by Arabic numerals, while different subfamilies are represented by capital letters from A to Q [4]. For instance, CYP1B2 refers to CYP family 1, subfamily B and protein 2. There are more than 13,000 *CYP* genes representing more than 400 families among various species [5] The number of *CYP* genes varies greatly among different organisms [6,7,8,9]. A total of 57 *CYP* genes have been identified in human, belonging to 18 families and 44 subfamilies [6]. Similarly, 54 genes encoding CYPs have been identified in Japanese pufferfish (*Takifugu rubripes*) [9]. In addition, about 246 and 328 *CYP* genes were also found from the genomes of *Arabidopsis* and rice, respectively [7]. The difference in the number of *CYP* genes in different organisms may be the result of adaptation to diverse environments [10]. Moreover, this *CYP* polymorphism may be related to drug metabolic capacity and risk of disease development [11].

CYPs can be classified into different categories based on different views. Traditionally, some CYPs are involved in the detoxification of xenobiotics, and others are involved in the biosynthesis of endogenous compounds. Eukaryotic CYPs are mainly located in the endoplasmic reticulum (ER) bound to the membrane, while prokaryotic ones participate in the electron transfer process in the cytosol [12,13]. In addition, some mitochondrial membrane-binding CYPs have been found [14]. Structurally, CYP proteins usually contain several conserved motifs and substrate recognition sites [15,16]. X-ray crystal structure and molecular dynamics simulations (MDS) show that some active sites of the CYP proteins have obvious flexibility [17]. This conformational change increases the interaction between the active sites of CYP proteins and various compounds.

CYPs catalyze the oxidative metabolism of both xenobiotics and endogenous compounds, such as drugs, pesticides, chemical carcinogens, hormones, phytochemicals, and environmental contaminants, etc. [1]. Moreover, production of a vast diversity of plant natural products, such as steroids and terpenoids, is based on the function of CYPs [18]. Some tumorigenesis also has been associated with the *CYP* genes [19]. In fish, expression patterns of some *CYP* family genes could be changed by various compounds, such as pesticides, benzopyrene, polychlorinated biphenyls, and 4-tert-pentylphenol [20,21,22,23,24]. Furthermore, some receptors, such as the aryl hydrocarbon (Ah) receptor, constitutive and rostane receptor (CAR), pregnane X receptor (PXR), and retinoic X receptor (RXR) are involved in the binding of xenobiotics and the promoter regions of *CYPs*, thus regulating the expression of the *CYP* genes [25].

Over the past 30 years, organophosphorus pesticides (OPs) have accelerated agricultural production. They can bind to cholinesterase on the synaptic membrane and prevent its catalytic action. Accumulated acetylcholine can then overexcite insects and eventually lead to death [26]. At the same time, the uncontrolled use of OPs has also brought environmental pollution problems. When entering water, OPs have a serious impact on some aquatic organisms [27]. OPs widely interfere with physiological processes, including enzyme activity, oxidative metabolism, and osmotic regulation, and exert immunotoxic effects on organisms [28,29]. Dimethoate (O,O-dimethyl S-(N-methylcarbamoylmethyl)-dithiophosphate) is one kind of the OPs widely used in experiments. An important biological resource and the first animal taxon with both innate and adaptive immune systems, studies of fish not only deepen our understanding of the evolution of the vertebrate immune system, but also help to strengthen environmental monitoring and protect an important food supply. Some *CYPs* have been considered as reliable biomarkers for monitoring pollutants in aquatic animals [30,31]. *Pungitius sinensis*, also known as Amur stickleback, belongs to the family Gasterosteidae. Because of its biological characteristics like strong vitality, small size, and short reproductive cycle, this fish will be an important model system for many researchers. Threespine stickleback (*Gasterosteus aculeatus*) especially has a wide range of applications in physiological and behavioral studies. Compared with *G. aculeatus*, the research on *P. sinensis* is limited. This study first identified 58 putative *CYP* genes based on the transcriptome sequencing data of Amur stickleback. Next, motif distribution, phylogeny, recombination, selective pressure, and expression profiles were performed to explore their evolutionary relationships. This study will be helpful for further functional studies of this gene family.

## 2. Materials and Methods

### 2.1. Materials, Organophosphorus Pesticide Exposure, and RNA Sequencing and Analysis

*P. sinensis* were obtained from aquaculture farms. Each mock and experimental group consisted of three fish hold separately at 22–23 °C. After 2 days of environmental adaptation, dimethoate (C5H12NO3PS2) was added to the experimental group at a 2.1 mg/L concentration for 24 h. The mock group was composed of fish cultured in freshwater. In these experiments, the entire fish body was used for RNA extraction. The RNA samples were pooled from three biological replicates. An equimolar amount of RNA was used for RNA sequencing. The Illumina Hiseq2000 platform was used to sequence the library at Shanghai OE Biotech Co., Ltd. (Shanghai, China). In transcriptome sequencing analysis, the expression level of Unigene can be estimated by counting the sequence reads. Reads counts were positively correlated with gene length and sequencing depth, as well as the true expression level of the gene. Using the database, the expression abundance of each Unigene in each sample was identified by sequence similarity alignment. Bowtie 2 [32] was used to obtain the reads number on Unigene, and eXpress [33] was used to calculate the FPKM (fragments per kilobase of transcripts per million fragments) value in each sample. The FPKM value was used to indicate the expression level of each transcript [34,35].

### 2.2. Identification of Putative CYP Proteins in the P. sinensis

To identify the putative CYP proteins in this RNA sequencing data, the Hidden Markov Model (HMM) profile of the conserved CYP domain (pfam00067) was used to perform a BLAST search against the Ensembl database [36] with an E-value cutoff of 1 × 10^−1^. Next, the CDD (a Conserved Domain Database) [37] and the Pfam databases [38] were used to further verify whether the candidates belong to this family. Some members that do not contain the conserved CYP domain are removed. ProtParam [39] and CELLO (subCELlular LOcalization predictor) [40] were used to predict the biochemical characteristics and the subcellular localization of these CYP proteins, respectively. Searching of the best matched sequence and species was performed on the National Center for Biotechnology Information database (NCBI) [41].

### 2.3. Phylogeny, and Conserved Motif Analysis of the CYP Gene Family

To explore the phylogenetic relationships among these CYP members, multiple sequence alignment was performed using the MUSCLE (MUltiple Sequence Comparison by Log-Expectation) method [42]. Next, a neighbor-joining phylogenetic tree was constructed using MEGA6 (Molecular Evolutionary Genetics Analysis) [43] with parameters: *p*-distance substitution model, 1000 bootstrap replications, and insertion of pairwise deletion gaps. Maximum likelihood (ML) method was also used to construct another phylogenetic tree with Jones–Taylor–Thornton (JTT) model, 100 bootstrap replications and partial deletion. Additionally, conserved motifs of these CYP proteins were identified using the MEME (Multiple Em for Motif Elicitation) program [44] with the following parameters: a maximum of eight motifs and 6–50 widths.

### 2.4. Recombination Events and Detecting of the CYP Genes

Potential recombination events at the *CYP* genes were identified using RDP (Recombination Detaction Program) v4.8 [45]. Five methods (RDP [46], GENECONV [47], MaxChi [48], Chimaera [48], and TOPAL [49]) were used to analyze the CDS (coding sequence) of the *CYP* genes with a *p*-value cutoff of 0.05 and 100 permutations.

### 2.5. Site-Specific Selection Assessment and Testing

Selective pressure at each residue of the CYP proteins was estimated using the value of the synonymous (*Ks*) and non-synonymous rates of nucleotide substitution (*Ka*). Four evolutionary models [M8 (beta+w ≥ 1), M8a (beta+w = 1), M7 (beta), and M5 (gamma)] were used to calculate the *Ka/Ks* values. Among these models, a Bayesian inference approach was used to represent how the characteristics evolved with probabilistic terms, and their biological assumptions were also used to assume a statistical distribution. M8 allows positive selection of proteins. A proportion p0 of the sites are drawn from a beta distribution, and a proportion p1 (=1 − p0) of the sites are drawn from an additional category beta+w (≥ 1). Therefore, sites drawn from the beta distribution are experiencing purifying selection, whereas those from the beta+w category are experiencing either neutral or positive selection. The M8a model is similar to the M8 model, but positive selection is not allowed by setting beta+w = 1. The M7 model is also similar to M8 except that it only assumes a beta distribution without additional category, which is mainly used for protein purifying selection. The M5 model assumes that *Ka/Ks* among sites are gamma distributed, and thus may allow for purifying, neutral, and positive selection [50]. All these models assume a statistical distribution to account for heterogenous *Ka/Ks* values among sites. The distributions are approximated using eight discrete categories following the previous study [51] and the *Ka/Ks* values are computed by calculating the expectation of the posterior distribution [50]. Comparison of these models allows statistical testing of the hypothesis of positive selection on protein (H1) by comparing the hypothesis with the null model (H0). LRT (likelihood ratio test) and AICc (Alaike Information Criterion) are allowed for the nested and no-nested models, respectively. The three-dimensional structure of the Psi_CYP4V protein in Group III was predicted with the Phyre2 server [52]. The effect of a point mutant on protein stability was predicted using I-Mutant2.0 [53].

## 3. Results and Discussion

### 3.1. Identification, Phylogenetic Analysis, and Motifs Distribution

A total of 58 putative *CYP* genes were identified in the *P. sinensis* genome (Table S1). They encode 183–557 amino acids with 5.50 to 9.57 pI values. Also, 91.4% of the predicted CYP proteins exhibit highly hydrophobic characteristics. Most CYPs were predicted to be localized in the plasma membrane (44.8%), mitochondrion (41.4%) or cytoplasm (36.2%) by CELLO [40].

**Figure 1:**
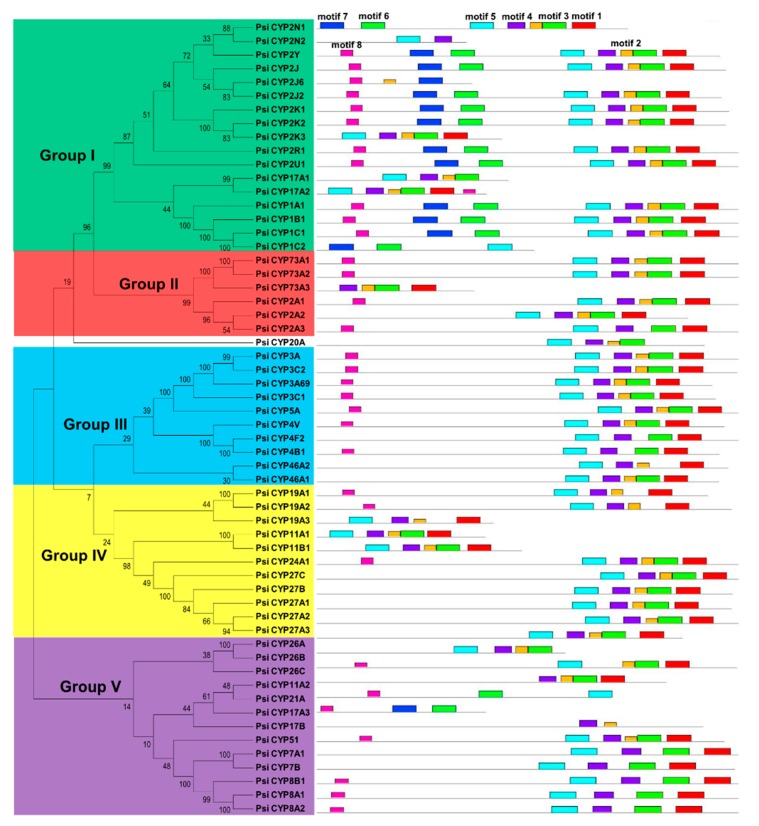
Phylogenetic relationships and motif compositions of the *CYP* genes in the *P. sinensis*. The phylogenetic tree was constructed and classified into five groups. Different motifs of the CYP proteins are displayed by different-colored boxes.

In order to evaluate the evolutionary relationship among *CYP* family genes in this fish, phylogenetic analysis was carried out based on a NJ (Neighbor-Joining) method. ML theory also was used to generate another phylogenetic tree (Figure S1), which had very similar topology with the NJ tree. Here, the NJ tree was selected for further analysis. These 58 CYP proteins were divided into five groups according to sequence similarity (Figure 1), although the bootstrap support was low for the most fundamental nodes of the tree. The motif distribution described below also supports this classification. The CYP1 family belongs to Group I, which is involved in the metabolism and activation of carcinogens in fish [54]. Its expression is induced by a variety of compounds such as pesticides, polychlorinated biphenyls, and benzopyrene. Therefore, CYP1 is often used as a biomarker for assessing aquatic environmental pollution [55,56]. The CYP2 family shows the largest degree of divergence, and except for CYP2A in Group II, other subfamilies (CYP2J, CYP2K, CYP2N, etc.) are in Group I (Figure 1). These CYP2 members catalyze foreign and endogenous compounds in fish [57]. CYP3 and CYP4 were assigned to Group III. The CYP3 family shows testosterone hydroxylase activity, and the enzyme activity of some members is induced by treatment with dexamethasone, dehydroepiandrosterone (DHEA), and other compounds [58,59]. The CYP4 family catalyzes the hydroxylation of fatty acids, and some peroxisome proliferating agent can induce its transcription [60]. One family of Group IV, CYP19, catalyzes C19 androgen to form aromatic C18 estrogen. It is the terminal steroid-producing enzyme in the estrogen biosynthesis pathway, and therefore plays a role in temperature-dependent sex determination [61]. Retinoic acid (RA) plays an important role in embryonic development of vertebrates [62]. CYP26 family enzymes from Group V metabolize RA to hydroxylated polar derivatives [63]. Deletion of CYP26 genes in zebrafish can lead to severe damage of the hindbrain [62].

**Figure 2:**
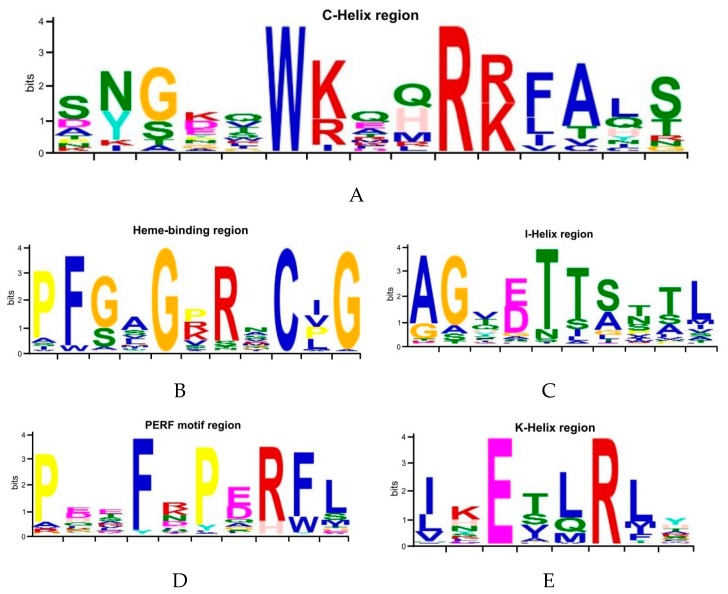
Motif and variation patterns among *P. sinensis CYP* genes. The height of a letter indicates its relative frequency at the given position for the amino acid. Five conserved regions are shown here: (**A**) C-helix region (WxxxR); (**B**) Heme-binding region (PFxxGxRxCxG/A); (**C**) I-helix region (GxE/DTT/S); (**D**) PERF motif region (PxxFxPE/DR); and (**E**) K-helix region (ExLR).

We also compared structural diversity with MEME [44]. Eight conserved motifs were found among these predicted CYP proteins (Figure 1). In general, most members have the same motif distribution within each group, which suggests the similarity of their functions [64,65]. In addition, several specific motifs were found within some groups. For example, motifs 6 and 7 were restricted to Group I. Among them, a conserved C-helix region (WxxxR) (Figure 2A) was located in motif 7 and its function was considered to neutralize the charge of propionate side chains of heme through tryptophan and arginine [66]. Moreover, others conserved heme-binding region (PFxxGxRxCxG/A), I-helix region (GxE/DTT/S), PERF motif region (PxxFxPE/DR), and K-helix region (ExLR) were found in motifs 1, 2, 3, and 4, respectively (Figure 2B–E). The heme-binding region exists in the C-terminal of CYP proteins, which forms a specific secondary structure and is involved in the NADPH-dependent electron transport pathway [67]. The I-helix region is responsible for proton transfer during monooxygenation. The K-helix region is essential for stabilizing the heme core structure of CYPs [68]. These conserved or variable regions play key roles in the functional divergence and complexity of CYP proteins.

### 3.2. Comparison of the CYP Genes among P. sinensis and Other Species

Next, we investigated the number of changes of the *CYP* genes among the *P. sinensis* and zebrafish (*Danio rerio*), medaka (*Oryzias latipes*), threespine stickleback (*Gasterosteus aculeatus*), Atlantic cod (*Gadus morhua*), green-spotted puffer (*Tetraodon nigroviridis*), and fugu puffer (*Takifugu rubripes*). The results indicated that a number of the *CYP* genes have undergone a great change following genome duplication in these fish species, and their expansion of the *CYP* gene family mainly occurred in Group I (Table 1; Figure S2). Fewer *CYP* genes exist in Groups III and IV of Atlantic cod, and in Group III of fugu puffer, respectively. Moreover, taxon-specific expansion of the *CYP* genes was found to occur in the Group II of *P. sinensis*. The comparative analysis revealed some evolutionary changes of the *CYP* gene family during the separation of these fish taxa. This family has undergone extensive gene gain and loss in evolution, and the gene duplication of *CYP* members in Group I was more significant. In zebrafish, we also examined their chromosome locations and found that over 56.9% of them were arranged in tandem, especially on chromosomes 20, 23, and 25. This suggests that the amplification of *CYP* genes in zebrafish may come from tandem duplication. In addition, synteny analysis of the *CYP* genes were also performed to demonstrate more detailed evolutional relationship in several fish and other species (Figure S3). There is a *CYP* gene cluster on chromosome 20 of zebrafish. However, most of these genes in the cluster have disappeared in threespine stickleback, Atlantic cod, and fugu puffer, suggesting that tandem duplication contributes the amplification of zebrafish *CYP* genes. In addition, we also found that some *CYP2J20*-like genes were duplicated in chicken, turkey, and mouse (Figure S3). Previous studies have indicated that the number of *CYP* genes varies greatly in different organisms. About 50–70 *CYP* genes have been identified in mammals [8]. Moreover, the number of *CYP* family genes has exceeded 200 in plants [7]. Similarly, in insects, the number of *CYP* genes in flour beetle *Tribolium castaneum* was 1.72 times higher than that of fruit fly *Drosophila melanogaster* [69,70]. The number variation of *CYP* genes may be the result of adaptation to ecological environment in different organisms [10].

### 3.3. Detection of Intragenic Recombination Events in the P. sinensis CYP Genes

**Figure 3:**
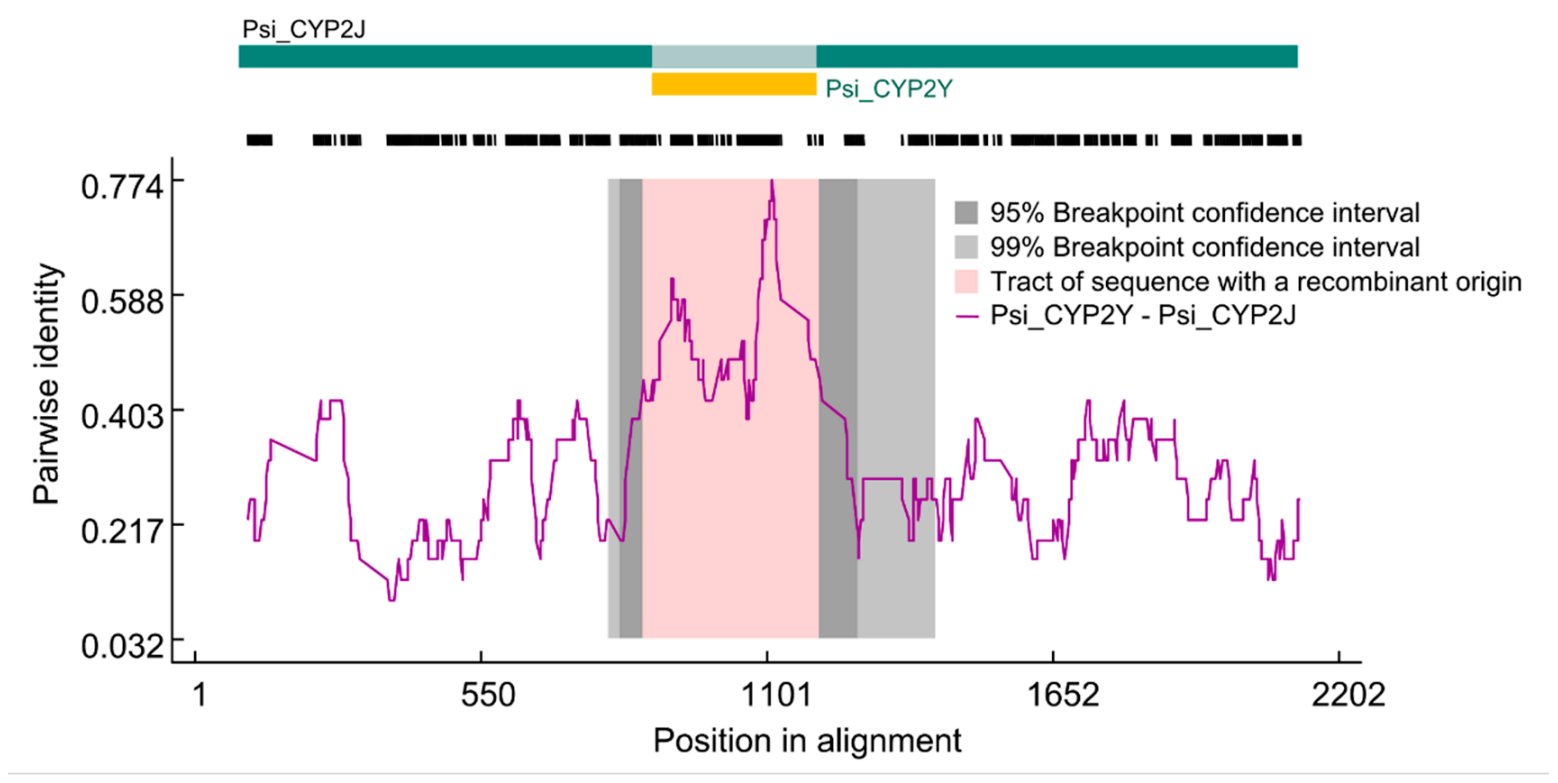
Identification of recombination events between the *Psi_CYP2J* and *Psi_CYP2Y* genes. Recombination events were inferred by the RDP (Recombination Detection Program) method. The broken black horizontal line indicates the sequence on hand for the genes at issue.

Recombination results in the exchange of sequences within or between genes, which affects the gene structure and genetic components [71,72]. To explore the evolutionary properties of *P. sinensis CYP* genes, five recombination methods (RDP, Geneconv, MaxChi, Chimaera, and TOPAL) embedded in the RDP v4.8 software [45] were used to investigate their recombination events. A total of 40 *CYP* genes were evaluated and they experienced 22 recombination events (Table 2). The MaxChi method detected more recombination signals than other methods due to its Maximum χ^2^ method in the data evaluation process [73,74]. A recombination event of *Psi_CYP2J* and *Psi_CYP2Y* is presented as an example (Figure 3). An obvious recombination signal appears in the central region of *Psi_CYP2J* gene. A previous study has demonstrated that *CYP* genes include multiple cases of interparalog chimeras, and the polymorphic *CYP12A4*/*CYP12A5* chimera correlates with resistance to an insecticide [10]. Our results suggest multiple *P. sinensis CYP* genes undergoing recombination events. Therefore, recombination increased the complexity of *P. sinensis CYP* genes and may have played important roles in functional divergence and genetic evolution of this gene family.

### 3.4. Selective Pressure at Amino Acid Sites of the CYP Members

**Figure 4:**
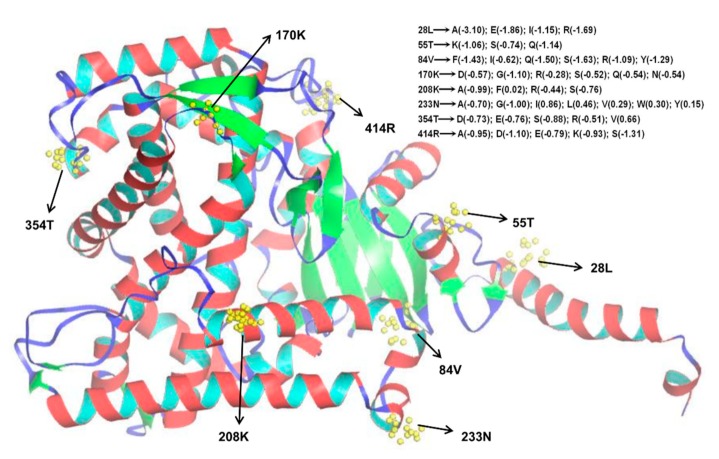
Distribution of positive selection sites among Group III CYP members predicted by the M5 model of nucleotide substitution.

Selective pressure of different amino acid sits was assessed using the *Ka*/*Ks* value, which indicates positive or purifying selection when greater or less than one, respectively [75]. Peptides experiencing selective pressure often alter the structure and hence the function of proteins during evolution. Duplicated genes usually have three destinies: neo-functionalization, sub-functionalization, or pseudogenization. Among them, the neo-functionalization and sub-functionalization are under positive and purifying selection, respectively [76,77,78]. To explore which peptides had undergone selection in evolution, the *Ka*/*Ks* value of each CYP site was calculated (Table 3). Group V had higher *Ka*/*Ks* values than the other groups, indicating that the changes among these members are relatively fast. All *Ka*/*Ks* values were below one, indicating that most *P. sinensis CYP* genes were subjected to purifying selection during evolution. In addition, several positive selection sites were identified in Group III and Group V under the M5 model (which assumes a gamma distribution over ω) (Table 3). As an example, the location of eight positive selection sites in Group III is described in detail (Figure 4; Figure S3). Among them, three sites (28L, 208K, and 354T) are located on the alpha helices (A, E, and J), respectively. Moreover, the 208K site was also located on substrate recognition site 2 (SRS2) (Figure S1). SRSs are responsible for the identification and binding of metabolic substrates. Here, a residue (208K) in SRS2 was positively selected, suggesting that the type of substrate recognized may change. We also found that positive selection site (414R) was located near the PERF motif region, suggesting that the 414R site may affect the function of this domain.

Next, I-Mutant2.0 software [53] was used to evaluate the effect of mutants of these positive selecting sites on folding stability of the protein. As a result, over 82.5% of mutants at these sites relaxed the stability of the CYP protein (Figure 4), which makes the protein less stable, but also may promote the functional diversification of CYP members. Previous studies have also indicated that positive selection induced the functional diversity of some CYP members [79,80,81]. It was consistent with the variety of CYP functions, including essential metabolism, developmental regulation, and pollutant defense [6]. Moreover, some positive selection sites are associated with substrate-binding regions, similar to our results described above. Of the nine positive selection sites identified in a previous study [80], two homologous sites (55T and 208K) were also found in this study (Figure 4; Figure S4). These findings suggest that some *CYP* genes are subject to natural selection to change their peptides for coping with xenobiotic exposure in evolution. It is an adaptation of organisms to environmental changes.

### 3.5. Expression Profiles of the P. sinensis CYP Genes under Dimethoate Stress Based on Transcriptome Data

**Figure 5:**
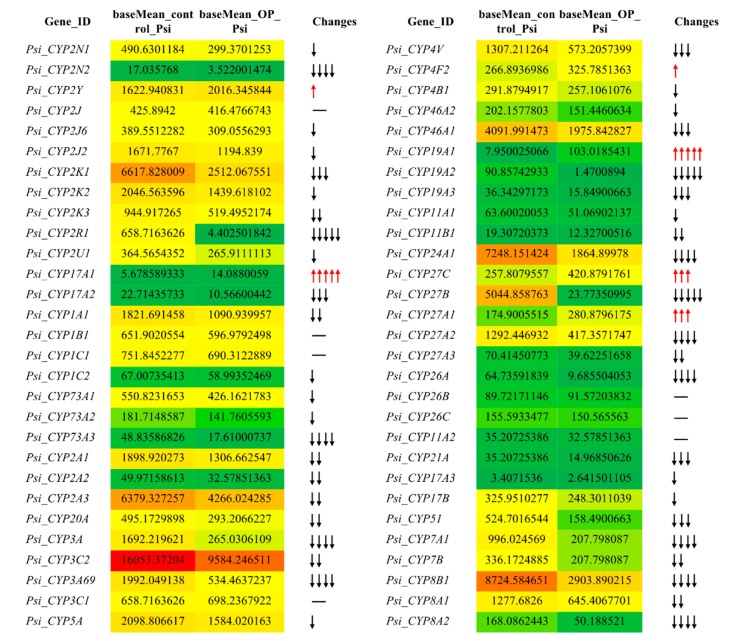
Expression profile changes of the *P. sinensis CYP* genes following dimethoate treatment.

To verify whether the *P. sinensis CYP* genes are also affected by dimethoate stress, their expression patterns were first investigated under this pesticide stress using the transcriptome data. The results indicated that expression levels of 77.59% and 10.34% of the *P. sinensis CYP* genes decreased or increased, respectively, under dimethoate stress (Figure 5). Among them, compared with the control group, expression levels of the *Psi_CYP27B* and the *Psi_CYP2R1* genes decreased about 212.2 and 149.6 times following the dimethoate treatment, respectively. Meanwhile, expression levels of the *Psi_CYP19A1* gene increased about 12.9 times under the same condition. CYP19A1 catalyzes the terminal step in the conversion of androgens to estrogens, and therefore plays a role in sex determination. Previous studies have indicated that CYP19A1 mRNA is increased or suppressed with a temperature-dependent manner in the liver of females or males, respectively, suggesting its involvement in sexual development [61,82]. Moreover, inhibition of aromatase activity can cause sex reversal in fish [83,84]. Some environmental pollutants, such as pesticides, have been shown to affect sex conversion of fish [85,86]. Here, dimethoate treatment can improve the transcriptional level of the *Psi_CYP19A1* gene, suggesting a potential effect of sex conversion.

We also investigated the response of some duplicated *CYP* genes, such as *Psi_CYP2N1/Psi_CYP2N2*, *Psi_CYP2K1/Psi_CYP2K2/Psi_CYP2K3*, *Psi_CYP17A1/Psi_CYP17A2*, *Psi_CYP19A1/Psi_CYP19A2/Psi_CYP19A3*, and *Psi_CYP8A1/Psi_CYP8A2*, to the dimethoate stress, and found significantly differences among them (Figure 5). As a family of membrane-bound hemoproteins, CYPs are involved in the detoxification and cellular metabolism of xenobiotics [1]. Furthermore, by altering neuroimmune communication, OPs can target several molecules of the immune system and play an immunotoxic effect [87]. Here, the expression patterns of most *P. sinensis CYP* genes were affected by dimethoate stress, suggesting that these *CYP* genes may be involved in the detoxication of OPs.

## 4. Conclusions

This study provided a comparative analysis of the *CYP* gene family in *P. sinensis*, which were classed into five groups by phylogenetic analyses. Motif compositions of the CYPs were highly conserved within each group, indicative of their functional conservation. Intragenic recombination plays a key role in the evolution of *CYP* genes. A few sites associated with the functional divergence were identified via selection analysis. Differential expression patterns of the *CYP* genes provided insights into possible functional divergence under dimethoate treatment. This study will provide a useful reference for further functional analysis of this gene family.

## Figures and Tables

**Table 1:** Variation in number of *CYP* genes among *P. sinensis* and other fish species.

Group	*P. sinensis*	*D. rerio*	*O. latipes*	*G. aculeatus*	*G. morhua*	*T. nigroviridis*	*T. rubripes*
I	17	34	18	15	18	18	10
II	6	0	0	0	0	0	0
III	10	9	8	7	8	9	3
IV	11	8	8	9	4	8	7
V	13	12	9	11	3	11	7
Others	1	1	1	1	0	1	1

**Table 2:** Predicted recombination events among *CYP* genes in *P. sinensis*.

Event Number	Found In	Recombination	Major Parent	Minor Parent	Detection Methods				
					RDP	GENECONV	MaxChi	Chimaera	TOPAL
1	1	*Psi_CYP26C*	Unknown	*Psi_CYP26A*	-	-	+	+	+
2	1	*Psi_CYP2U1*	*Psi_CYP2J*	Unknown	-	-	+	-	-
3	1	*Psi_CYP27A2*	*Psi_CYP27C*	Unknown	-	-	+	-	-
4	1	*Psi_CYP2K2*	*Psi_CYP2J*	Unknown	-	-	+	-	-
5	1	*Psi_CYP7B*	*Psi_CYP8B1*	*Psi_CYP26C*	-	-	+	-	-
6	1	*Psi_CYP2K1*	*Psi_CYP2J*	Unknown	-	-	+	-	+
7	2	*Psi_CYP3A*	*Psi_CYP46A*	Unknown	-	-	+	-	-
8	1	*Psi_CYP1B1*	*Psi_CYP2A1*	Unknown	-	-	+	-	-
9	1	*Psi_CYP2K2*	*Psi_CYP2J*	*Psi_CYP2A1*	-	-	+	-	-
10	1	*Psi_CYP24A1*	Unknown	*Psi_CYP5A*	-	-	+	-	-
11	1	*Psi_CYP27A3*	*Psi_CYP27*	*Psi_CYP24A1*	-	-	+	-	-
12	1	*Psi_CYP27A3*	*Psi_CYP3C1*	Unknown	-	-	+	-	-
13	2	*Psi_CYP73A1*	*Psi_CYP2K1*	*Psi_CYP3A69*	-	+	+	-	-
14	1	*Psi_CYP73A1*	*Psi_CYP17A1*	Unknown	-	-	+	-	-
15	1	*Psi_CYP11A2*	*Psi_CYP27B*	*Psi_CYP73A2*	-	-	+	-	-
16	1	*Psi_CYP3A69*	*Psi_CYP26A*	*Psi_CYP17B*	-	-	+	-	-
17	1	*Psi_CYP17A1*	*Psi_CYP2N1*	*Psi_CYP17A3*	-	-	+	-	-

**Table 3:** Likelihood values and parameter estimates regarding selection upon *CYP* genes.

Branches	Models	*Ka/Ks*	Log-Likelihood	Numbers of PSS *
Group I	M8 (beta+w ≥ 1)	0.2757	−20,905.7	0
	M8a (beta+w = 1)	0.2781	−20,904.8	0
	M7 (beta)	0.2600	−20,893.9	0
	M5 (gamma)	0.3149	−20,940	0
Group II	M8 (beta+w ≥ 1)	0.2859	−8912.75	0
	M8a (beta+w = 1)	0.2879	−8913.55	0
	M7 (beta)	0.2752	−8909.22	0
	M5 (gamma)	0.3308	−8947.28	0
Group III	M8 (beta+w ≥ 1)	0.3429	−17,468.6	0
	M8a (beta+w = 1)	0.3455	−17,469.5	0
	M7 (beta)	0.3291	−17,465.5	0
	M5 (gamma)	0.4137	−17,517.7	8
Group IV	M8 (beta+w ≥ 1)	0.3083	−15,652.2	0
	M8a (beta+w = 1)	0.3177	−15,663.2	0
	M7 (beta)	0.2983	−15,654.2	0
	M5 (gamma)	0.3534	−15,694.7	0
Group V	M8 (beta+w ≥ 1)	0.4936	−20,366.6	0
	M8a (beta+w = 1)	0.4937	−20,367.1	0
	M7 (beta)	0.4767	−20,363.7	0
	M5 (gamma)	0.6040	−20,454.8	35

* PSS: positive selection site.

## Supplementary Materials

The following are available online at https://www.mdpi.com/2076-2615/9/11/873/s1, **Table S1.** *CYP* genes identified in this study. **Figure S1.** Phylogenetic tree generated by ML method. **Figure S2.** Phylogenetic analysis of *CYP* genes in Amur stickleback and other six fish species. **Figure S3.** Synteny analysis of *CYP* genes in several fish and other species. The position of *CYP* genes and neighboring genes on chromosomes are shown. **Figure S4.** Sequence alignment of the Group III *CYPs* in *P. sinensis*. Sequence alignment was performed by using ClustalW. Five conserved regions (C-Helix region, I-Helix region, K-Helix region, PERF motif region, and heme-binding region) are written in red. The 12 alpha helices are marked (from A to L) with green lines. Six substrate recognition sites (SRS1–6) are boxed with blue rectangles. Eight positive selective sites are presented in red background.
