# Supplementary material for: Transcriptome-Based Identification and Molecular Evolution of the Cytochrome P450 Genes and Expression Profiling under Dimethoate Treatment in Amur Stickleback (Pungitius sinensis)
Source: Animals (Basel). 2019 Oct 28;9(11):873. doi: 10.3390/ani9110873 (PMC6912322; doi:10.3390/ani9110873)
Supplement: Supplementary file 1 [file animals-09-00873-s001.zip › animals-535551-supplementary files for publication/Fig. S4.pdf]

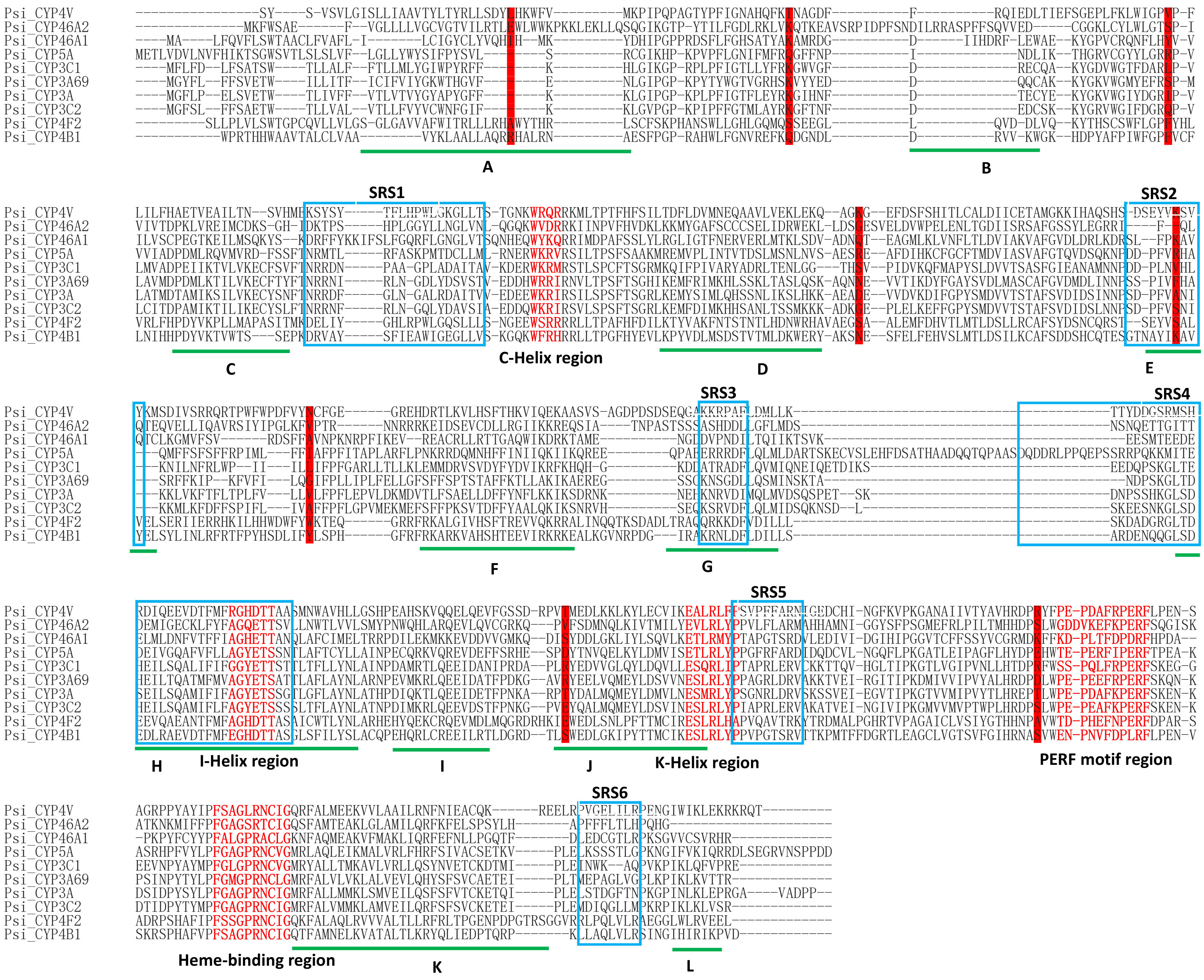
Psi_CYP4V
Psi_CYP46A2
Psi_CYP46A1
Psi_CYP5A
Psi_CYP3C1
Psi_CYP3A69
Psi_CYP3A
Psi_CYP3C2
Psi_CYP4F2
Psi_CYP4B1
A
B
SRS1
SRS2
C
C-Helix region
D
E
SRS3
SRS4
F
G
SRS5
H
I-Helix region
I
J
K-Helix region
PERF motif region
SRS6
Heme-binding region
K
L
