# Supplementary figures and images for: Transcriptome-Based Identification and Molecular Evolution of the Cytochrome P450 Genes and Expression Profiling under Dimethoate Treatment in Amur Stickleback (Pungitius sinensis)
Source: Animals (Basel). 2019 Oct 28;9(11):873. doi: 10.3390/ani9110873 (PMC6912322; doi:10.3390/ani9110873)

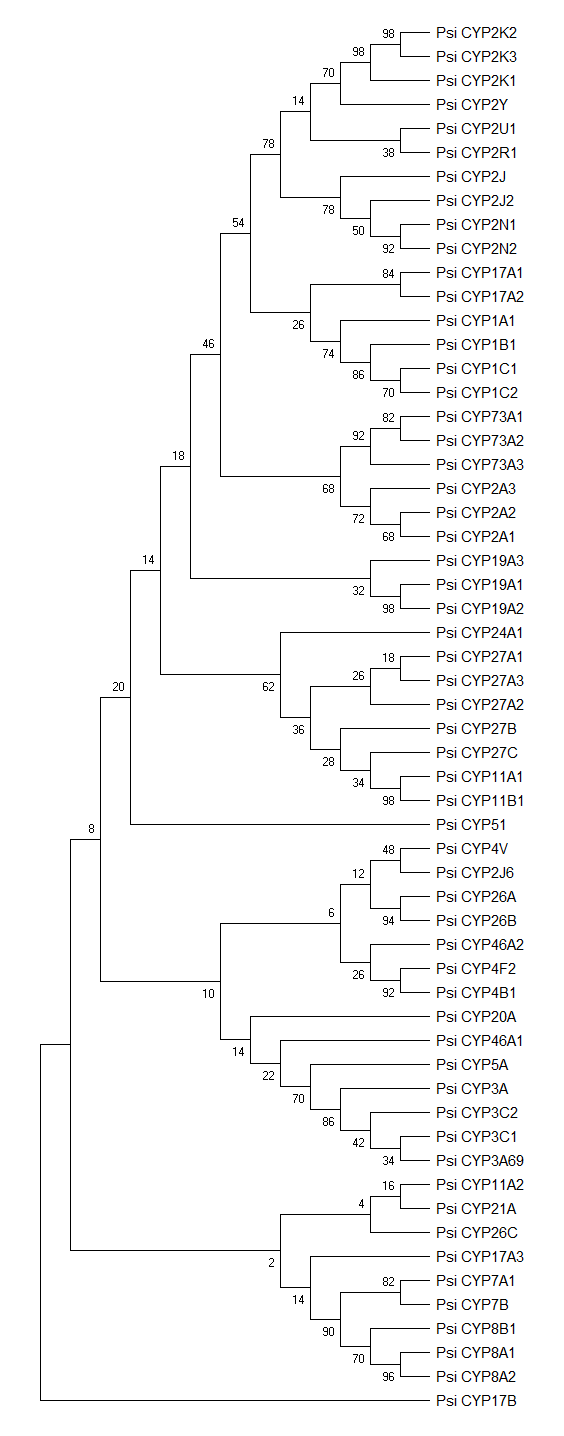

Supplement: Supplementary file 1 [file animals-09-00873-s001.zip › animals-535551-supplementary files for publication/Fig. S1 ML-tree.png]

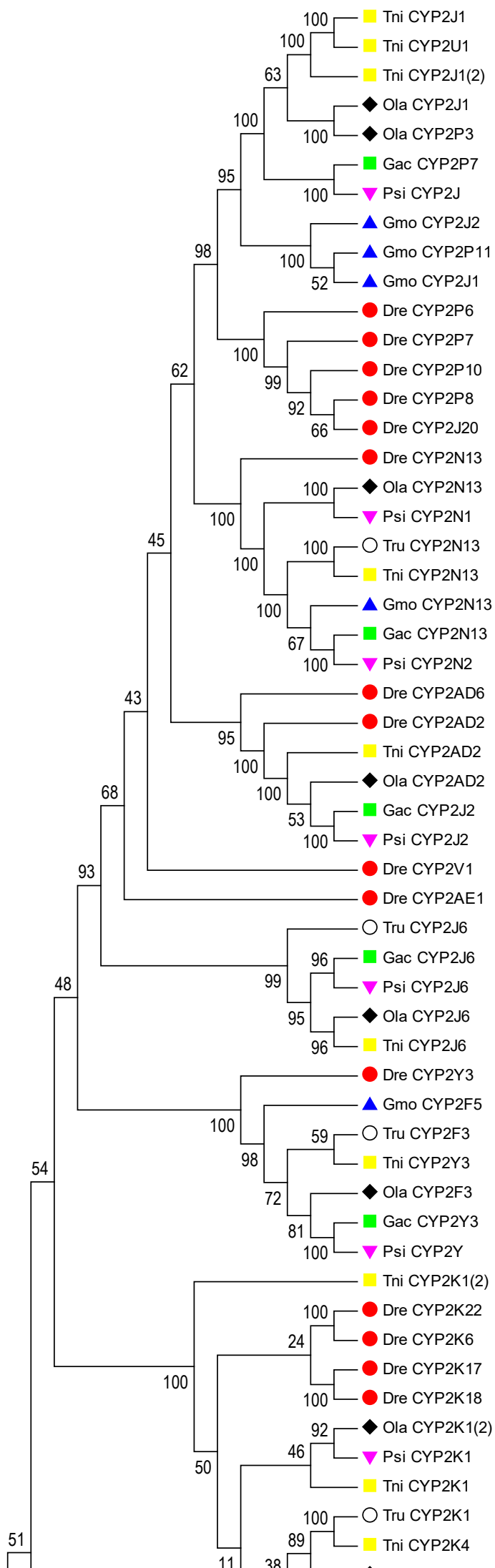

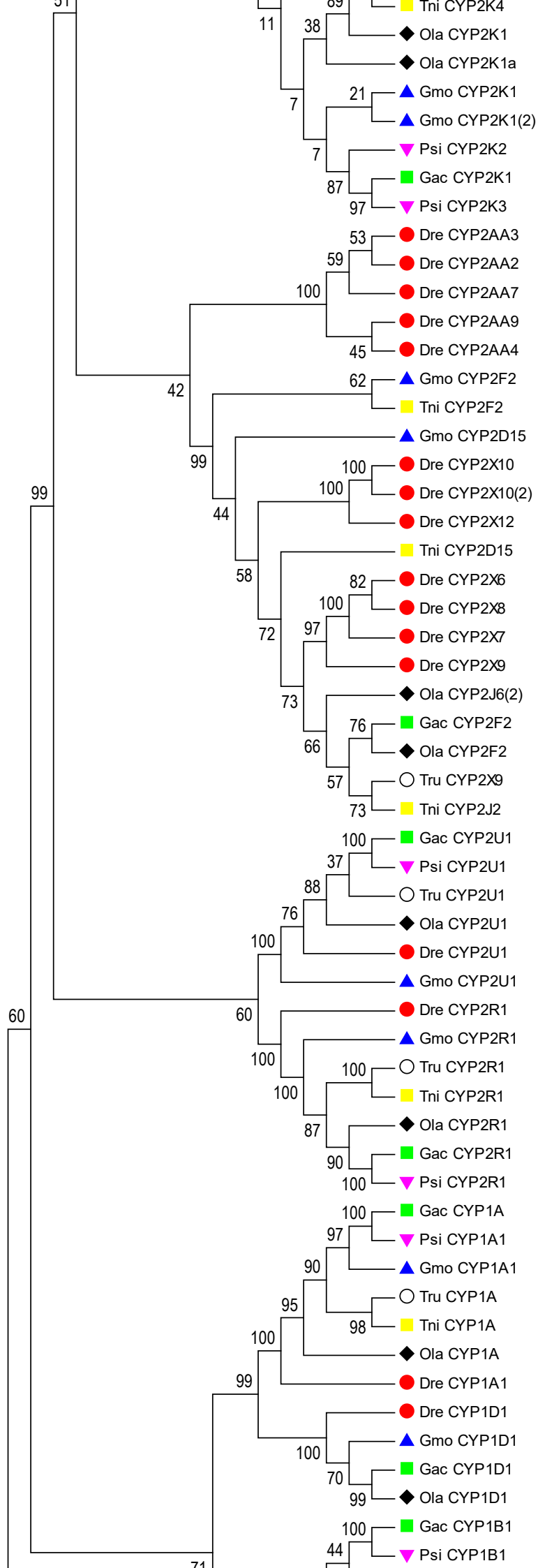

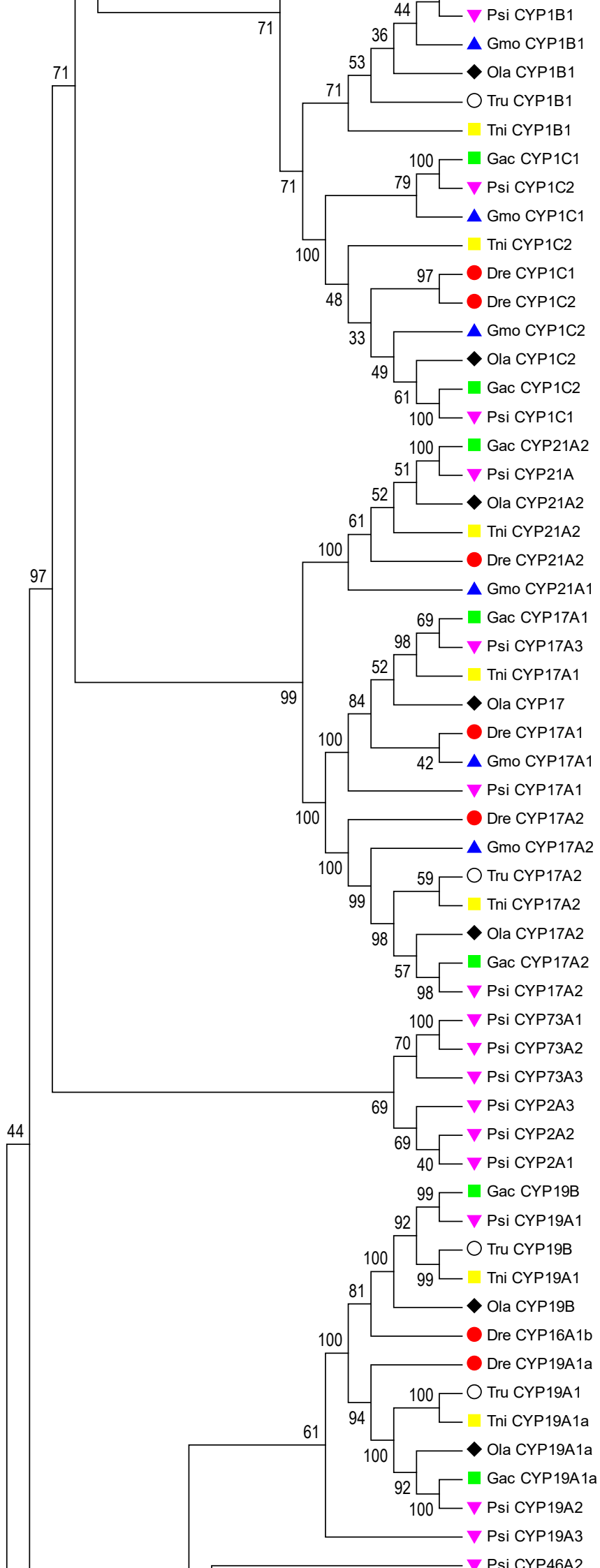

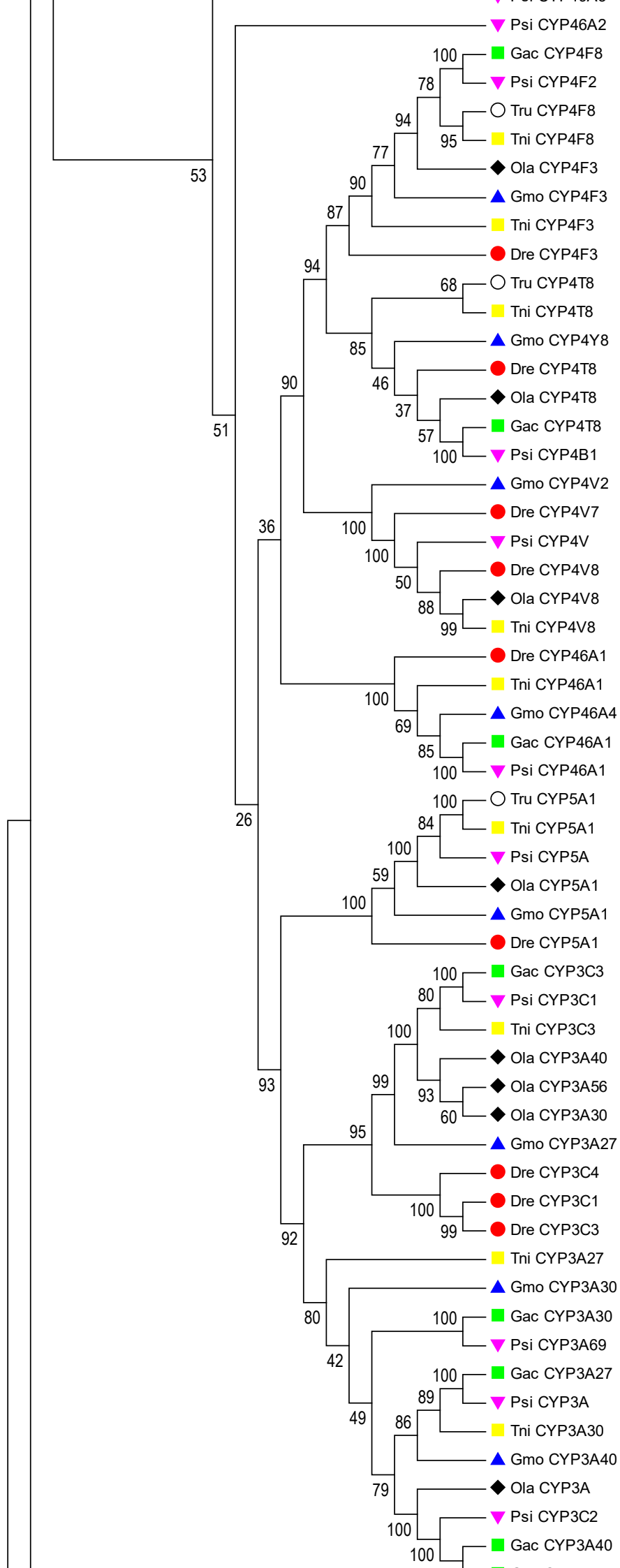

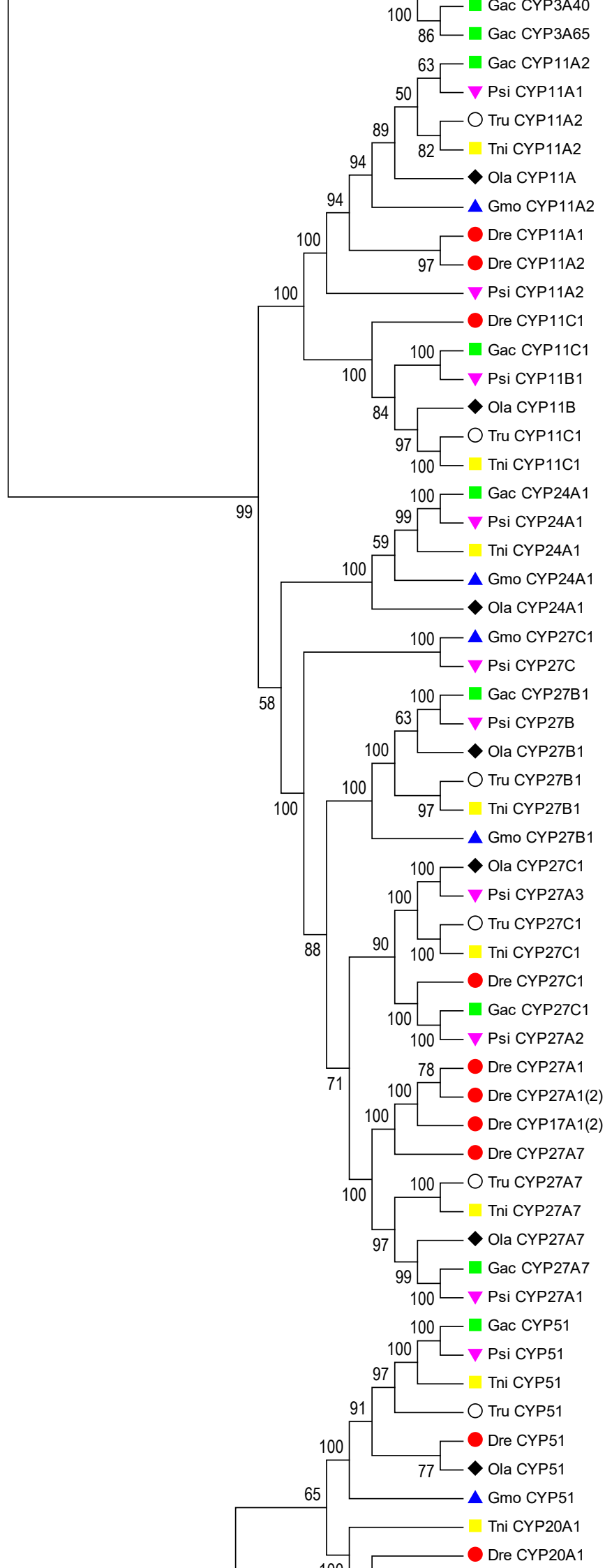

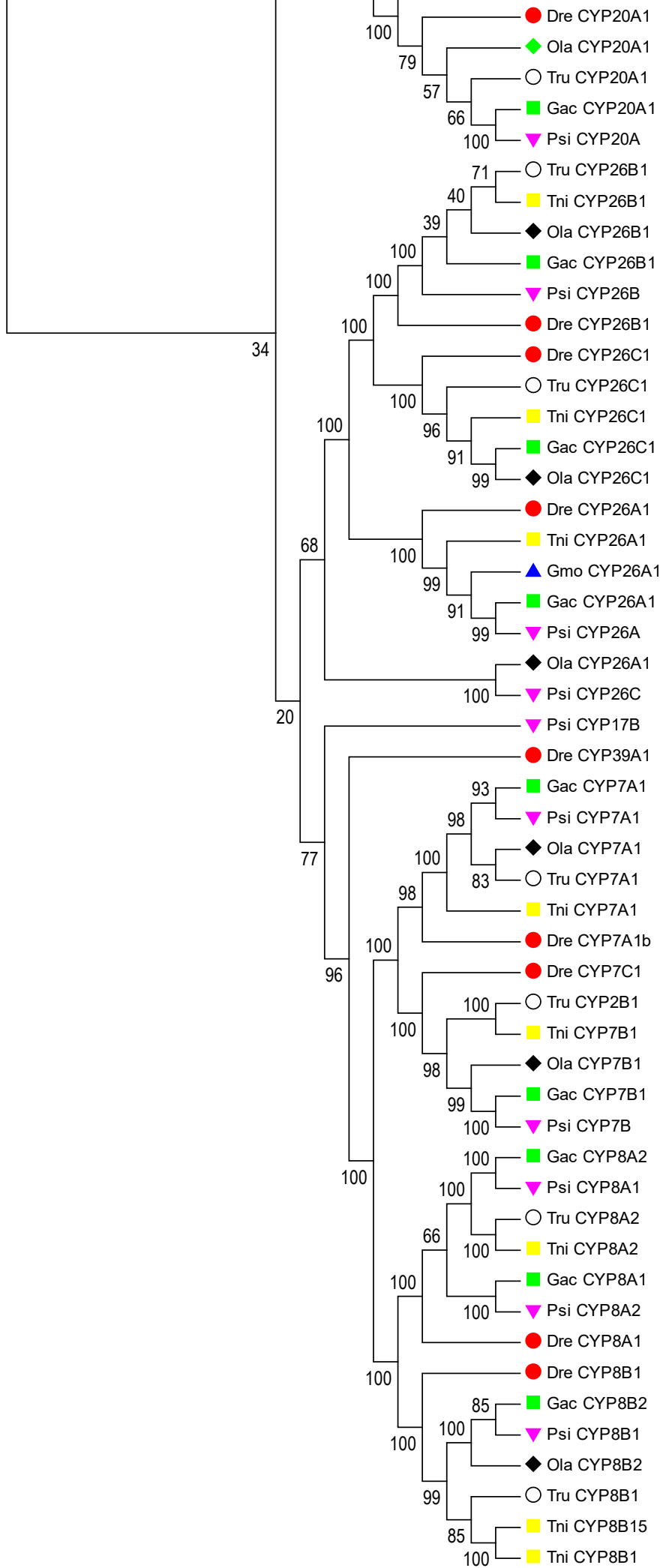

Supplement: Supplementary file 1 [file animals-09-00873-s001.zip › animals-535551-supplementary files for publication/Fig. S2.PDF]

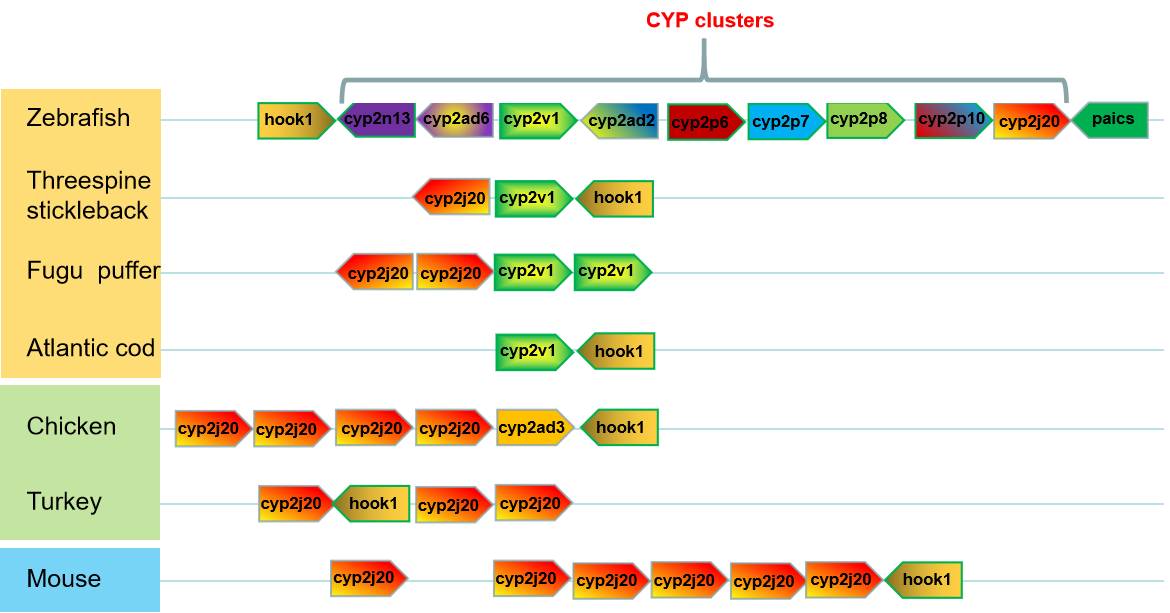

Supplement: Supplementary file 1 [file animals-09-00873-s001.zip › animals-535551-supplementary files for publication/Fig. S3.png]
