# Supplementary material for: Transcriptome-Based Identification and Molecular Evolution of the Cytochrome P450 Genes and Expression Profiling under Dimethoate Treatment in Amur Stickleback (Pungitius sinensis)
Source: Animals (Basel). 2019 Oct 28;9(11):873. doi: 10.3390/ani9110873 (PMC6912322; doi:10.3390/ani9110873)
Supplement: Supplementary file 1 [file animals-09-00873-s001.zip › animals-535551-supplementary files for publication/animals-535551-supplementary Table S1 for publication.doc]

Supplementary files: Transcriptome-Based Identification and Molecular Evolution of the *Cytochrome P450* Genes and Expression Profiling under Dimethoate Treatment in Amur Stickleback (*Pungitius sinensis*)

Jun Cao * and Xiuzhu Cheng

**Table S1.** *CYP* genes identified in this study.

| **Name** | **Protein Length** | **pI** | **Mw (KD)** | **GRAVY** | **CELLO predictions** | **Matched sequence ID** | **Matched species** | **E-value** | **Identities** |
| --- | --- | --- | --- | --- | --- | --- | --- | --- | --- |
| Psi_CYP4V | 507 | 8.05 | 58.063 | −0.301 | Cytoplasmic (1.147) PlasmaMembrane (1.142) Mitochondrial (1.035) | XP_018558733.1 | *Lates calcarifer* | 0 | 412/506 (81%) |
| Psi_CYP8A1 | 485 | 7.64 | 55.084 | −0.276 | Extracellular (2.634) | XP_028432274.1 | *Perca flavescens* | 0 | 395/485 (81%) |
| Psi_CYP5A | 557 | 8.66 | 64.189 | −0.202 | PlasmaMembrane (3.034) | XP_028441643.1 | *Perca flavescens* | 0 | 491/562 (87%) |
| Psi_CYP3A | 507 | 5.50 | 57.504 | −0.131 | PlasmaMembrane (2.101) | XP_028462148.1 | *Perca flavescens* | 0 | 401/506 (79%) |
| Psi_CYP27A2 | 523 | 8.82 | 59.292 | −0.139 | Mitochondrial (2.472) | XP_026233129.1 | *Anabas testudineus* | 0 | 421/524 (80%) |
| Psi_CYP2N1 | 379 | 6.21 | 42.92 | −0.247 | Cytoplasmic (2.840) | XP_027146361.1 | *Larimichthys crocea* | 0 | 313/378 (83%) |
| Psi_CYP2J | 498 | 6.04 | 56.585 | −0.144 | PlasmaMembrane (1.470) Cytoplasmic (1.193) Extracellular (1.109) | XP_028442367.1 | *Perca flavescens* | 0 | 375/498 (75%) |
| Psi_CYP3C1 | 496 | 8.36 | 56.829 | −0.058 | PlasmaMembrane (3.032) | XP_028459748.1 | *Perca flavescens* | 0 | 363/497 (73%) |
| Psi_CYP1B1 | 525 | 9.14 | 59.264 | −0.082 | PlasmaMembrane (1.441) Mitochondrial (1.298) | ADO15702.1 | *Gasterosteus aculeatus* | 0 | 482/525 (92%) |
| Psi_CYP73A1 | 504 | 9.1 | 57.988 | −0.279 | Cytoplasmic (2.422) | XP_010921158.1 | *Elaeis guineensis* | 0 | 421/503 (84%) |
| Psi_CYP2J6 | 190 | 9.14 | 21.22 | 0.027 | Extracellular (1.727) PlasmaMembrane (1.690) | XP_019207044.1 | *Oreochromis niloticus* | 0 | 141/190 (74%) |
| Psi_CYP20A | 461 | 6.29 | 51.044 | −0.089 | Cytoplasmic (1.828) | XP_028427664.1 | *Perca flavescens* | 0 | 413/459 (90%) |
| Psi_CYP7A1 | 513 | 7.71 | 58.958 | −0.239 | Mitochondrial (2.050) | AIT82984.1 | *Dicentrarchus labrax* | 0 | 476/513 (93%) |
| Psi_CYP1C1 | 524 | 6.45 | 58.339 | −0.053 | Cytoplasmic (1.483) | ADO15704.1 | *Gasterosteus aculeatus* | 0 | 474/524 (90%) |
| Psi_CYP17B | 471 | 6.62 | 52.702 | −0.195 | Cytoplasmic (1.611) | KMZ61450.1 | *Zostera marina* | 0 | 469/471 (99%) |
| Psi_CYP27C | 540 | 8.33 | 60.909 | −0.165 | Mitochondrial (1.851); Cytoplasmic (1.368) | XP_028437642.1 | *Perca flavescens* | 0 | 470/541 (87%) |
| Psi_CYP7B | 471 | 8.78 | 53.828 | −0.224 | Mitochondrial (1.675) Cytoplasmic (1.316) PlasmaMembrane (1.124) | XP_020481523.1 | *Labrus bergylta* | 0 | 355/472 (75%) |
| Psi_CYP4F2 | 524 | 9.01 | 60.232 | −0.291 | Extracellular (1.852) Mitochondrial (1.509) | XP_028427755.1 | *Perca flavescens* | 0 | 432/522 (83%) |
| Psi_CYP2U1 | 527 | 8.38 | 58.821 | 0.019 | PlasmaMembrane (3.756) | XP_019110856.2 | *Larimichthys crocea* | 0 | 418/528 (79%) |
| Psi_CYP4B1 | 501 | 8.97 | 57.689 | −0.324 | Mitochondrial (1.387) PlasmaMembrane (1.025) | XP_028435777.1 | *Perca flavescens* | 0 | 402/501 (80%) |
| Psi_CYP26C | 426 | 8.21 | 47.754 | −0.256 | Extracellular (2.292) | XP_028457112.1 | *Perca flavescens* | 0 | 374/426 (88%) |
| Psi_CYP51 | 496 | 6.78 | 56.286 | −0.217 | Mitochondrial (1.685) | XP_028453536.1 | *Perca flavescens* | 0 | 466/497 (94%) |
| Psi_CYP2R1 | 517 | 8.59 | 58.783 | −0.115 | PlasmaMembrane (2.254) | XP_010729681.1 | *Larimichthys crocea* | 0 | 478/517 (92%) |
| Psi_CYP1C2 | 260 | 6.20 | 29.761 | −0.392 | Nuclear (1.203) Mitochondrial (1.102) | NP_001254621.1 | *Gasterosteus aculeatus* | 2 × 10^-174^ | 238/260 (92%) |
| Psi_CYP27A1 | 505 | 9.00 | 57.992 | −0.225 | Mitochondrial (2.511) | XP_018552157.1 | *Lates calcarifer* | 0 | 402/516 (78%) |
| Psi_CYP8A2 | 481 | 8.91 | 55.158 | −0.427 | Mitochondrial (1.369) | XP_028432190.1 | *Perca flavescens* | 0 | 404/485 (83%) |
| Psi_CYP73A2 | 505 | 9.19 | 58.006 | −0.243 | Cytoplasmic (2.508) | KMZ62158.1 | *Zostera marina* | 0 | 503/505 (99%) |
| Psi_CYP46A2 | 512 | 8.81 | 58.257 | −0.086 | PlasmaMembrane (2.486) | KMZ60604.1 | *Zostera marina* | 0 | 508/512 (99%) |
| Psi_CYP27A3 | 446 | 8.81 | 50.678 | −0.261 | Mitochondrial (2.429) | XP_018516582.1 | *Lates calcarifer* | 0 | 331/460 (72%) |
| Psi_CYP17A1 | 234 | 5.99 | 26.162 | −0.197 | Cytoplasmic (2.713) | NP_001254585.1 | *Gasterosteus aculeatus* | 4 × 10^-159^ | 225/235 (96%) |
| Psi_CYP11A1 | 214 | 8.71 | 24.581 | −0.055 | Mitochondrial (2.321) | AWP08401.1 | *Scophthalmus maximus* | 2 × 10^-142^ | 202/214 (94%) |
| Psi_CYP19A1 | 487 | 8.36 | 55.18 | 0.009 | PlasmaMembrane (3.382) | XP_028439955.1 | *Perca flavescens* | 0 | 427/487 (88%) |
| Psi_CYP2K2 | 498 | 8.05 | 57.036 | −0.209 | PlasmaMembrane (3.210) | XP_026181102.1 | *Mastacembelus armatus* | 0 | 311/497 (63%) |
| Psi_CYP26A | 304 | 6.46 | 34.785 | −0.34 | Cytoplasmic (3.036) | XP_028423608.1 | *Perca flavescens* | 0 | 286/304 (94%) |
| Psi_CYP2J2 | 493 | 6.00 | 56.23 | −0.171 | PlasmaMembrane (2.020) | XP_023260191.1 | *Seriola lalandi dorsalis* | 0 | 399/493 (81%) |
| Psi_CYP17A2 | 207 | 6.53 | 22.989 | −0.056 | PlasmaMembrane (2.533) | NP_001254584.1 | *Gasterosteus aculeatus* | 5 × 10^-132^ | 190/207 (92%) |
| Psi_CYP73A3 | 189 | 6.67 | 21.367 | −0.243 | Cytoplasmic (2.058) Mitochondrial (1.578) | KMZ73659.1 | *Zostera marina* | 9 × 10^-133^ | 189/189 (100%) |
| Psi_CYP2A2 | 441 | 8.52 | 50.944 | −0.338 | Cytoplasmic (1.656) Mitochondrial (1.438) Nuclear (1.429) | KMZ71833.1 | *Zostera marina* | 0 | 269/441 (61%) |
| Psi_CYP11A2 | 298 | 9.41 | 34.532 | −0.364 | Mitochondrial (2.006) | XP_028441535.1 | *Perca flavescens* | 0 | 270/299 (90%) |
| Psi_CYP19A2 | 516 | 7.96 | 58.063 | −0.102 | PlasmaMembrane (1.722) Extracellular (1.128) | AGH69789.1 | *Anoplopoma fimbria* | 0 | 458/516 (89%) |
| Psi_CYP21A | 361 | 6.52 | 40.403 | −0.071 | PlasmaMembrane (2.142) | XP_019110682.1 | *Larimichthys crocea* | 0 | 293/361 (81%) |
| Psi_CYP19A3 | 224 | 7.75 | 25.722 | −0.318 | Cytoplasmic (1.429) Mitochondrial (1.018) | KMZ62148.1 | *Zostera marina* | 6 × 10^-162^ | 222/224 (99%) |
| Psi_CYP11B1 | 259 | 9.57 | 28.559 | −0.224 | Mitochondrial (2.897) | XP_028435951.1 | *Perca flavescens* | 2 × 10^-166^ | 238/265 (90%) |
| Psi_CYP26B | 511 | 8.97 | 57.66 | −0.118 | Mitochondrial (1.611) | AZI94876.1 | *Lutjanus guttatus* | 0 | 502/512 (98%) |
| Psi_CYP2N2 | 183 | 6.08 | 20.672 | −0.161 | Cytoplasmic (2.105) Mitochondrial (1.568) | XP_023120685.1 | *Amphiprion ocellaris* | 7 × 10^-109^ | 153/183 (84%) |
| Psi_CYP17A3 | 208 | 8.78 | 22.933 | 0.048 | PlasmaMembrane (1.150) | NP_001254585.1 | *Gasterosteus aculeatus* | 8 × 10^-134^ | 194/210 (92%) |
| Psi_CYP3C2 | 499 | 8.64 | 57.192 | −0.082 | PlasmaMembrane (2.340) | XP_028449449.1 | *Perca flavescens* | 0 | 442/499 (89%) |
| Psi_CYP8B1 | 511 | 9.20 | 58.948 | −0.304 | Mitochondrial (1.692)  Cytoplasmic (1.586) | XP_023259505.1 | *Seriola lalandi dorsalis* | 0 | 412/510 (81%) |
| Psi_CYP2K1 | 502 | 8.60 | 56.91 | −0.219 | PlasmaMembrane (1.569) | XP_028460253.1 | *Perca flavescens* | 0 | 414/502 (82%) |
| Psi_CYP24A1 | 513 | 8.42 | 58.484 | −0.294 | Cytoplasmic (2.891) | TDH13588.1 | *Perca flavescens* | 0 | 484/513 (94%) |
| Psi_CYP2A3 | 509 | 7.25 | 58.389 | −0.218 | Cytoplasmic (1.789) | KMZ74076.1 | *Zostera marina* | 0 | 504/509 (99%) |
| Psi_CYP46A1 | 500 | 7.85 | 57.518 | −0.126 | Cytoplasmic (1.283) PlasmaMembrane (1.002) | XP_028460909.1 | *Perca flavescens* | 0 | 395/500 (79%) |
| Psi_CYP2Y | 491 | 8.67 | 56.282 | −0.135 | PlasmaMembrane (2.581) | XP_023271856.1 | *Seriola lalandi dorsalis* | 0 | 394/491 (80%) |
| Psi_CYP2K3 | 226 | 5.98 | 25.427 | −0.14 | Extracellular (1.407) | XP_022608317.1 | *Seriola dumerili* | 1 × 10^-120^ | 164/226 (73%) |
| Psi_CYP27B | 506 | 8.90 | 57.789 | −0.172 | Mitochondrial (3.044) | XP_018554656.1 | *Lates calcarifer* | 0 | 462/511 (90%) |
| Psi_CYP3A69 | 492 | 8.60 | 56.126 | −0.043 | PlasmaMembrane (3.415) | XP_010789073.1 | *Notothenia coriiceps* | 0 | 364/497 (73%) |
| Psi_CYP2A1 | 515 | 8.68 | 58.055 | 0.012 | PlasmaMembrane (1.883) Cytoplasmic (1.329) | KMZ68049.1 | *Zostera marina* | 0 | 447/515 (87%) |
| Psi_CYP1A1 | 521 | 6.61 | 59.052 | −0.136 | PlasmaMembrane (1.094) Cytoplasmic (1.035) | ADO15701.1 | *Gasterosteus aculeatus* | 0 | 497/521 (95%) |
